# TNF Production and Release from Microglia via Extracellular Vesicles: Impact on Brain Functions

**DOI:** 10.3390/cells9102145

**Published:** 2020-09-23

**Authors:** Stefano Raffaele, Marta Lombardi, Claudia Verderio, Marta Fumagalli

**Affiliations:** 1 Department of Pharmacological and Biomolecular Sciences, Università degli Studi di Milano, 20133 Milan, Italy; stefano.raffaele@unimi.it; 2 CNR Institute of Neuroscience, 20129 Milan, Italy; marta.lombardi@in.cnr.it (M.L.); c.verderio@in.cnr.it (C.V.)

**Keywords:** microglia, extracellular vesicles, tumor necrosis factor (TNF), neuroinflammation, neuronal plasticity, remyelination

## Abstract

Tumor necrosis factor (TNF) is a pleiotropic cytokine powerfully influencing diverse processes of the central nervous system (CNS) under both physiological and pathological conditions. Here, we analyze current literature describing the molecular processes involved in TNF synthesis and release from microglia, the resident immune cells of the CNS and the main source of this cytokine both in brain development and neurodegenerative diseases. A special attention has been given to the unconventional vesicular pathway of TNF, based on the emerging role of microglia-derived extracellular vesicles (EVs) in the propagation of inflammatory signals and in mediating cell-to-cell communication. Moreover, we describe the contribution of microglial TNF in regulating important CNS functions, including the neuroinflammatory response following brain injury, the neuronal circuit formation and synaptic plasticity, and the processes of myelin damage and repair. Specifically, the available data on the functions mediated by microglial EVs carrying TNF have been scrutinized to gain insights on possible novel therapeutic strategies targeting TNF to foster CNS repair.

## 1. Introduction

Microglia represent the resident immune cells of the central nervous system (CNS). In physiological conditions they are involved in sensing the environment and regulating brain homeostasis by performing different functions. Upon brain injury, they transform from immunosurveillant into reactive cells and acquire a variety of immune-phenotypes committed to both detrimental and protective functions [1,2]. Reactive microglia express inflammatory molecules including antigen presenting receptors (MHCII, CD40), scavenger receptors, and lysosomal receptor CD68 [3,4], and release cytokines, free radicals, or trophic factors contributing to tissue injury or repair [5]. However, emerging data revealed that microglia, despite being extremely dynamic and plastic in their response, display intrinsic heterogeneity, with a huge diversity of subtypes specialized in specific tasks [6]. In addition, recent single-cell transcriptomic studies highlighted the existence of distinct microglial clusters in neurodegenerative and demyelinating diseases, that are totally absent in healthy conditions [7,8]. Interestingly, these pathology-associated microglial subpopulations are strictly found within CNS lesions and seem to be primed to sustain an inflammatory response, as they exclusively express a battery of cytokines, including tumor necrosis factor (TNF) [8,9,10].

Among the different factors through which microglia exert their regulatory activity on brain functions, TNF is the one that probably best reflects the dichotomous nature of these immune cells, being able to induce harmful or beneficial effects, depending on the specific isoform and receptor subtypes it activates. Indeed, while TNF has been historically defined as a potent inflammatory mediator driving tissue degeneration in disease conditions, growing evidence provides concrete clues of a parallel role of the cytokine in regenerative processes [11], thus complicating the use of TNF inhibitors in experimental models and clinical trials.

Here, we review current literature on the mechanisms regulating TNF production, intercellular trafficking and release from microglial cells, with a special attention to the role of microglia-derived extracellular vesicles (EVs) in these processes. Furthermore, we describe the variety of biological effects exerted by microglia-released TNF on different brain functions, which could be particularly relevant for the development of specific therapeutic strategies aimed at containing the progression of brain damage or promoting tissue repair.

## 2. TNF Expression, Signaling, and Release from Microglia

TNF is a pleiotropic cytokine produced as a precursor membrane-anchored form of 26 kDa, called transmembrane TNF (tmTNF), by immune cells, including microglia, astrocytes as well as other different cell types [12]. After cleavage of tmTNF by the metalloproteinase TNF converting enzyme (TACE/ADAM17), a soluble form (solTNF) of 17 kDa is generated and released into the extracellular space. Both soluble and transmembrane forms of TNF mediate a variety of physiological and pathological functions, through the activation of Types 1 and 2 TNF receptors (TNFR1 and TNFR2) [11]. SolTNF selectively binds and signals through TNFR1, while tmTNF can result in both TNFR1 and TNFR2 activation. Due to their different binding affinities, the cellular processes activated by the two receptors are often opposite: TNFR1 primarily mediates apoptosis and chronic inflammation [13], conversely, TNFR2 promotes cell survival, resolution of inflammation, immunity and repair [14,15]. The deleterious or beneficial effects depend on the activation of complex intracellular signaling pathways [16].

Upon TNF binding, TNFR1 recruits the TNFR1-associated death domain protein (TRADD) that induces cell apoptosis by activating Fas-associated death domain protein (FADD) and caspase 8 [17]. However, TRADD can recruit TNFR-associated factor 2 (TRAF2) and receptor-interacting protein kinase-1 (RIPK-1), leading to the activation of the transcription factors nuclear factor-κB (NF-κB) and AP-1, thus preventing cell death and promoting inflammatory response [18,19].

The intracellular signaling pathways activated by TNFR2 are less characterized compared with those of TNFR1. Given that TNFR2 lacks death domain, the receptor only recruits TRAF2 and activates PI3K-Akt/PKB and NF-κB pathways for mediating pro-survival signals [20,21]. Accordingly, it has been demonstrated that the activation of PI3K-Akt/PKB pathway upon TNFR2 stimulation in astrocytes leads to enhanced expression and secretion of several neuroprotective factors, including CXCL12 and leukemia inhibitory factor (LIF), which promote oligodendrocyte proliferation, differentiation and myelination [22].

Several studies showed that TNF is upregulated in activated microglia at transcriptional and translational level [23], in particular upon exposure to the cytokine interferon gamma (IFN-γ) [24], and it is itself capable of regulating microglia reaction [25]. Indeed, in vitro experiments showed that TNF enhances BV2 microglial activation by upregulating the NF-κB signaling pathway as well as by enhancing the expression of inflammatory mediators and cytokine production, including interleukin (IL)-6, IL-1β, and intercellular adhesion molecule-1 (ICAM-1) [25,26]. By contrast, treatment with TNF inhibitors reduces lipopolysaccharide (LPS)-induced microglial activation and indirectly affects production of cytokines and chemokines [27,28]. In vivo, both neuroinflammatory and neurodegenerative models develop TNF-secreting microglia, whose abundance correlates with the extent of neuroinflammatory response in mice affected by experimental autoimmune encephalomyelitis (EAE) [29], a typical model of multiple sclerosis (MS).

Increased release of TNF by microglia can enhance the activation of the entire population of microglia in a positive feedback mechanism [30,31,32], sustaining tissue degeneration (see Section 4).

In line with these findings, a large body of evidence indicated that microglial TNF is involved in brain damage following acute injury or chronic neuroinflammation [11]. High levels of TNF have been found in cerebrospinal fluid (CSF) and serum of patients and animal models of CNS pathologies, including cerebral stroke, trauma, Parkinson’s disease (PD), Alzheimer’s disease (AD), MS, and amyotrophic lateral sclerosis (ALS) [25,33,34,35,36]. Importantly, TNF neutralizing antibodies and inhibitors have been tested in patients affected by these disorders for reducing inflammation [37,38], and were the first biological drugs for rheumatoid arthritis [39].

Under pathological events, microglia and damaged cells release at the sites of CNS injury a large amount of ATP, a danger signal that activates specific receptors, called P2 purinoceptors, ubiquitously expressed on neurons and glia [40,41]. ATP is considered an important regulator of microglia functions [42,43], including cytokine release. The pioneer work by Hide and colleagues showed that ATP induces de novo synthesis and secretion of TNF from rat microglia through the activation of ionotropic P2X7 receptor, extracellular signal regulated kinase (ERK), c-Jun N-terminal kinase (JNK) and p38 mitogen-activated protein (MAP) kinase [44]. In response to ATP, P2X7 receptor induces a long-lasting Ca^2+^ influx in microglia [40] that evokes TNF secretion in a concentration-dependent manner [44]. However, P2X7-dependent TNF release occurs through a complex and still unknown mechanism, given that treatment of microglia with specific P2X7 antagonists does not affect its release [45].

MAP kinase cascades (ERK, JNK and p38 MAP kinase) are involved in TNF production downstream of P2X7 activation [46]. Specifically, ERK and JNK regulate TNF transcription by activating two transcriptional factors, the nuclear factor of activated T cells (NFAT) and the p65 subunit of NF-κB (RelA) [47], whereas p38 controls TNF post-transcriptional modification and nucleocytoplasmic transport by regulating the expression of RNA binding proteins [48].

**Figure 1:**
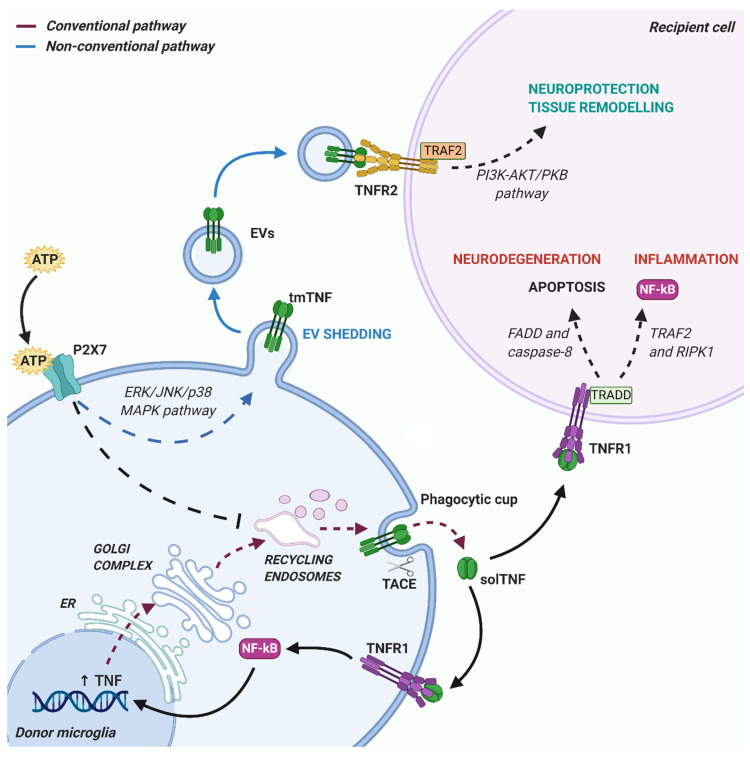
Schematic representation of the conventional and non-conventional pathways of tumor necrosis factor (TNF) release from microglia. The conventional pathway (purple dashed lines) requires cytokine localization in the endoplasmic reticulum (ER), where it is correctly folded and transported to Golgi complex for final post-translational modifications. TNF is then exposed on cell membrane (transmembrane TNF (tmTNF)) in the phagocytic cup, where it is cleaved by TNF converting enzyme (TACE) and released as a soluble form of TNF (solTNF) in the extracellular space. Released solTNF is able to interact with TNFR1 receptor on recipient cells and induce pro-inflammatory and neurodegenerative effects, or to bind TNFR1 receptors on donor microglial cells, creating a positive feedback loop reinforcing TNF production. The non-conventional release pathway (blue lines) instead consists in tmTNF membrane localization via an ER—Golgi independent route, avoiding post-translational modifications. In this case, bearing outside of the phagocytic cup and distant from TACE enzymes, tmTNF is protected from cleavage and can be packed into shedding EVs. Thus, EV-carried tmTNF preferentially interacts with TNFR2 receptors on target cells, triggering protective responses. Interestingly, ATP-mediated activation of P2X7 receptors on donor microglial cells is able to influence these mechanisms, inhibiting the conventional release pathway and favoring non-conventional TNF release into EVs. Created with BioRender.com.

Once synthetized, TNF is released via conventional or non-conventional pathways (Figure 1). The conventional pathway requires a rapid transcription and translation of the tmTNF, that contains the signal peptide targeting it to the endoplasmic reticulum (ER). In ER, the protein is correctly folded and then transported to the Golgi complex for final modifications [49]. Newly synthesized tmTNF converge at the trans-Golgi network (TGN), a specific compartment of the Golgi apparatus responsible for sorting cargoes to different carriers and routes [50]. The cytokine is sorted into tubular or vesicular carriers, which move along microtubules and are enriched in SNARE complex proteins, that mediate tmTNF transport to the plasma membrane [49]. In macrophages, the peripheral microglia counterparts, that SNARE complex and additional membrane proteins regulate the focal fusion of TNF-loaded recycling endosome with highly specific sites of the cell surface, called phagocytic cups [51]. This mechanism allows two advantages during infection: the expansion of macrophage membranes to engulf microbes and the specific delivery of tmTNF to a membrane structure enriched in TACE proteins (the phagocytic cup) where tmTNF is rapidly cleaved and released as active solTNF [52]. Importantly, a recent study suggested that intracellular TNF trafficking is rate-limiting for classical cytokine secretion in both macrophages and dendritic cells [53]. Like other constitutively secreted cytokines, TNF is able to self-regulate its secretion by signaling within recycling endosomes while in transit to the plasma membrane. Once released, TNF can be endocytosed by cells and within the endocytic compartments can continue to signal inside recipient cells [53]. These regulatory mechanisms are important in immune cells, including microglia, for increasing cytokine production in response to pathogenic stimuli, or limiting it during tissue repair.

The non-conventional secretory pathways typically mediate release of proteins lacking leader sequences, through an ER-Golgi transport-independent mechanism. Unconventional secretion allows cells to secrete cytokines without the requirement for post-translational modification, which can influence their functions, and may protect the host when conventional mechanisms are compromised, for example under ER stress [54]. Several routes for unconventional cytokine secretion have been described. These include cytokine interaction with the lipid membrane and formation of a permeable pore, membrane transporter routes [55] or cell death through pyroptosis [56]. In addition, unconventional vesicular pathways have been reported for many inflammatory proteins, such as galectin-3 (Gal-3), IL-1α, IL-18, fibroblast growth factor 2 (FGF2), and IL-1β, consisting in formation and shedding of membrane vesicles at the cell surface or vesicle budding inside endosomal compartments [54,57]. Despite having a signal peptide, TNF can be also released through unconventional vesicular pathways, in association with extracellular vesicles (EVs).

ATP is a major signal inducing unconventional TNF secretion via EVs [58]. In macrophages, ATP paradoxically inhibits the classical secretion of solTNF, favoring the unconventional release of tmTNFα in association with membrane vesicles [58].

## 3. TNF Release via Extracellular Vesicles (EVs)

As described above, TNF synthesis in microglia strictly depends on the activation state of these cells and it is finely regulated by signals released in the extracellular milieu in response to brain damage, including ATP [44]. Specifically, upon P2X7 receptor activation [59], ATP induces TNF release through an unconventional pathway consisting in the formation and release of EVs [60].

EVs are small particles formed by a double layer of phospholipids, which are gaining increasing interest as fundamental mediators of intercellular communications in the brain. EVs signals to adjacent cells but they can travel very long distances and deliver complex messages to distant cells [61]. EVs can transfer between cells not only hydrophilic components, such as cytokines and trophic factors, but also lipids, transmembrane proteins, mRNAs and miRNAs, which are protected from enzymatic degradation by the vesicular membrane [62]. Another important advantage of EVs-mediated signaling is the capacity to create a biologically active concentration of signaling molecules in close proximity to target cells, which could not induce any effect if diluted in the extracellular environment [63].

EVs can be classified based on site of origin in microvesicles (100 nm to 1 µm in diameter), directly shedding from the cell membrane, and exosomes (30–100 nm in diameter), originating from multivesicular bodies in the endosomal compartment [61]. Another classification system divides them in small and large EVs based on their dimension and other physical properties [64]. EVs interact with recipient cells with different mechanisms, including fusion, phagocytosis/pinocytosis and direct surface contact [62], suggesting that both membrane components and encapsulated factors may mediate biological effects. Interestingly, the composition of EVs strictly reflects the type and the activation state of donor cells, evoking, in the case of microglia, a detrimental or a beneficial response depending on the parental inflammatory or pro-resolving phenotype [65,66]. Due to these features, microglial EVs have been identified as mediators of inflammation and neurodegeneration, playing a pivotal role in spreading pathological misfolded protein aggregates [67,68,69,70] and in triggering harmful responses in recipient neurons and glial cells [71,72]. On the other hand, they have been also described as vehicles of pro-regenerative molecules, promoting remyelination and brain repair [69,72,73].

Given all the properties described above, EVs hold great potential for several research and clinical applications, ranging from the basic study of pathophysiological mechanisms [69], to the application as biomarkers of disease progression and patients response to treatment [74], or as vehicles for targeted delivery of therapeutic molecules [75]. However, some issues still need to be resolved in order to fulfill all these promises, including how to univocally purify all the different EV subtypes from biological fluids, to precisely enrich disease-relevant subsets for analysis or to selectively deliver EVs to target cells in vivo.

Nonetheless, EVs represent ideal carriers for the delivery of transmembrane proteins such as tmTNF. Intriguingly, EV production by microglia is massively induced by the same mechanism driving TNF expression, namely activation of the P2X7 receptor by ATP [60]. This supports the hypothesis that EV release may represent the main pathway for TNF secretion from ATP-activated microglia. Accordingly, recent studies demonstrated the presence of TNF, and other cytokines, in EVs produced from cultured microglia [72], monocytes and tissue explants [63]. Specifically, Fitzgerald and colleagues showed that more than 40% of the TNF released by monocytes is associated with EVs, and that the balance between TNF release mechanisms, free or EV-encapsulated, is affected by the activation state of donor cells [63]. Accordingly, a subsequent study showed that ATP stimulation profoundly influences the mechanisms of TNF release, shifting TNF secretion from the conventional release of solTNF to the preferential packaging of tmTNF in EVs [58]. Similar mechanisms have been described in EVs derived from microglial cells, where a significant increase in TNF levels was detected in response to inflammatory stimuli [72,76]. These findings are in line with the pioneer report by Verderio and colleagues, who first showed a role for microglial EVs in the spreading of pro-inflammatory signals [77].

Importantly, EVs can also carry solTNF among their cargoes and the TNF isoform, soluble versus membrane-bound, carried by EVs greatly impacts the response of recipient cells [76]. TmTNF is the prevalent form in EVs isolated from microglia upon stimulation with α-synuclein, to resemble neurodegenerative conditions of PD. In that case, EVs-associated tmTNF was shown to induce neuronal apoptosis probably via TNFR1 activation, an effect counteracted by anti-TNF antibodies [78]. However, given the association of tmTNF with protective effects through TNFR2 activation [79], a further characterization of the effects of EVs-associated tmTNF on neurons and other brain cells would be of great relevance to assess the possible protective role of TNF.

Besides TNF itself, other important players of the TNF signaling pathway have been reported in EVs, including TNFR1, associated signaling proteins and TACE, the enzyme responsible for tmTNF cleavage and release of solTNF from the plasma membrane. Indeed, TNFR1-containing small EVs have been detected both in vitro [80,81] and in circulating human plasma [82]. EV-mediated release of TNFR1 is a process regulated by cAMP-dependent protein kinase A (PKA) signaling [80] and depends on acid- and neutral-sphingomyelinases [81]. The production of EVs containing TNFR1 and associated TRADD proteins was described to be triggered by TNF itself as a feedback mechanism, capable to inhibit TNF-induced responses in parental cells and to prevent excessive inflammatory activity [81]. Furthermore, the release of TNF receptors through EVs may represent a mechanism of intercellular communication, as vesicular TNFR1 binding to tmTNF on the surface of target cells, acting in this case as a receptor, might activate a “reverse” signaling mechanism [83,84]. Indeed, tmTNF presents an intracellular domain containing a nuclear localization sequence of 10 kDa, which is able to detach upon tmTNF binding with soluble receptors [84] and to induce a specific anti-inflammatory response in tmTNF-bearing cells [85]. Interestingly, the same process can be activated also by the interaction between tmTNF and TNF-targeting drugs, i.e., monoclonal antibodies, crucially contributing to the positive outcome of the pharmacological treatment [86].

The presence of enzymatically active TACE within EVs has been reported in blood samples of HIV-infected subjects [87], whose release was mediated by an unconventional tyrosine-kinase regulated pathway [88]. In addition, EVs enriched in tmTNF and TACE were collected from the plasma of rats after induction of chronic cerebral ischemia. Interestingly these EVs were shown to induce apoptosis of endothelial cells in an in vitro model of blood brain barrier through activation of the TNF death pathway, leading to increased permeability [89]. However, since vesicular tmTNF has been reported to be protected against proteolytic cleavage [58], the enzymatic activity of EV-carried TACE may modulate solTNF levels in recipient cells, rather than mediating solTNF release directly from EVs.

As already mentioned, EVs containing TNF and related signaling molecules have been detected in the CSF [90] and in the human peripheral circulation [82,87,91], representing easily accessible biomarkers of neuroinflammation and neurodegeneration. Thus, it is increasingly clear that EVs may provide important information for the diagnosis of neurological disorders or as useful tools to monitor disease progression and patient’s response to pharmacological treatments [62,74,77,92]. However, more advances in the knowledge of the mechanisms determining the sorting of specific molecules into microglial EVs, as well as in the technologies used to analyze different types of EVs and their cargo in biological fluids, are still required to fully exploit the potential of EVs as reliable novel clinical biomarkers [93].

Collectively, the evidence described above suggests that microglia-derived EVs may represent ideal vectors for the delivery of TNF molecules, capable to promote both deleterious and reparative responses in target cells.

## 4. Impact of Microglia-Derived TNF on Brain Functions

**Figure 2:**
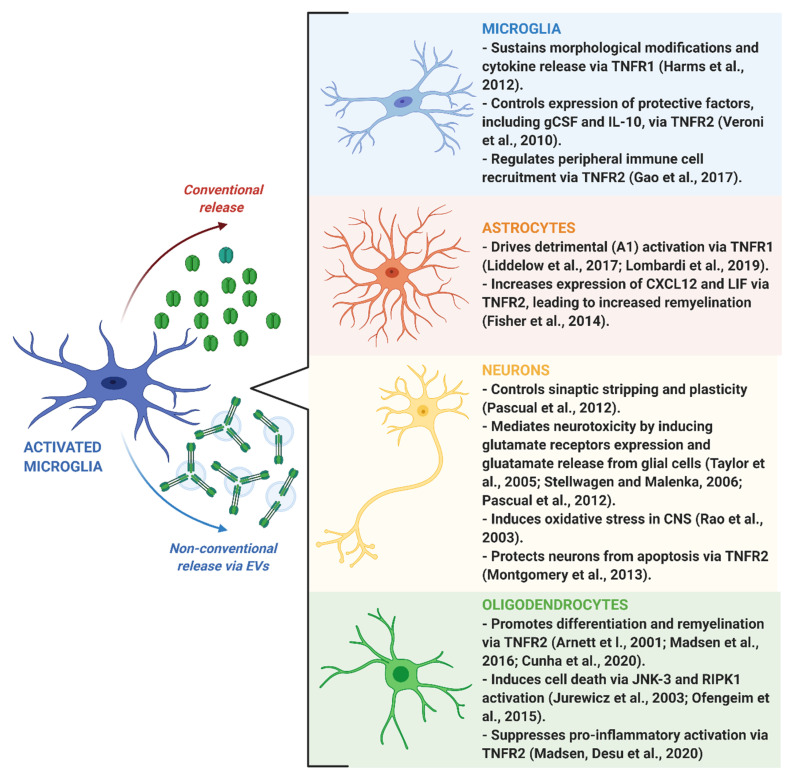
Schematic representation of the effects exerted by microglia-derived TNF on central nervous system (CNS) cells. Activated microglia is able to release TNF via conventional or extracellular vesicle (EV)-mediated non-conventional pathways. Once released, TNF induces specific detrimental or beneficial responses in recipient cells, including microglia, astrocytes, neurons and oligodendrocytes (right panels), through activation of TNFR1 and TNFR2 receptors. Created with BioRender.com.

As already discussed, inflammatory stimuli trigger a complex cellular and molecular response in microglial cells, which includes the release of soluble and transmembrane TNF and other related factors. In the following paragraphs, we will discuss the double-edged role of TNF deriving from microglial cells in shaping brain functions, including the neuroinflammatory response to tissue damage, neuronal circuit formation and synaptic plasticity, and myelin degeneration and repair (Figure 2).

### 4.1. Neuroinflammation

Neuroinflammation can be considered as the first line of defense to preserve or restore the CNS homeostasis in response to harmful stimuli, degenerative conditions and traumatic injuries. This process mainly involves the synchronized action of different cell types, primarily including microglia and astrocytes [94]. The progression of the neuroinflammatory response can be very different, depending on the heterogeneity of activation states acquired by microglia in response to different types of insult [2] and on the regional diversity of these cells, of which several subsets have been recently described, each committed to specific functions [6]. Recent evidence indicates the neuroinflammatory response be generally pro-resolving during the acute injury phase, whereas after disease chronicization it turns into a detrimental process, which hinders repair mechanisms and promotes degeneration [94].

Microglia-derived TNF has been implicated in sustaining the pro-inflammatory activation of microglial cells through a positive feedback mechanism mediated by TNFR1 activation [95]. Accordingly, results obtained in primary microglia isolated from TNF knockout mice demonstrated that autocrine stimulation by TNF is required for key effector functions exerted by microglia in response to inflammatory challenge, including morphological rearrangement and cytokine release, while TNF ablation did not affect microglia development, surveillance and phagocytic activity [96]. Interestingly, this autocrine process has been recently demonstrated to involve a microRNA induced by TNF, namely miR-342, which targets Bcl-2 associated anthanogene-1 (BAG-1), a protein that degrades NF-κB p65, thus sustaining NF-κB activation and consequent TNF release by microglia [32]. As a consequence, inhibiting miR--342 with specific drugs might be an effective strategy to reduce pathological microglia overactivation sustained by TNF [32].

In addition to the aforementioned effects on microglial cells, TNF released by inflamed microglia, together with IL-1α and C1q, has been demonstrated to drive detrimental A1 astrocyte activation, leading to a final toxic effect on neurons and oligodendrocytes underlying the pathological mechanisms of several neurodegenerative disorders [97]. Notably, the critical role of TNF in driving astroglial activation has been confirmed also in human astrocytes derived from induced pluripotent stem cells [98]. Recently, Lombardi and colleagues provided new details on the mechanism driving microglia-induced detrimental astrogliosis, demonstrating that this process is mediated by microglia-derived EVs carrying TNF [72]. Harmful A1 astroglial signature has been found also in the physiologically aged brain, and, interestingly, it was prevented in knockout mice with genetic ablation of the three microglial factors required for astrocytic A1 polarization, including TNF [99]. These results are coherent with a previously observed increase in basal TNF production by senescent microglia during aging [100], which may be involved in progressive cognitive impairment and predisposition to neurological disorders [101].

Similar to microglia, activated astrocytes themselves can produce and release TNF, giving rise to an autocrine/paracrine loop of activation and cytokine release [102]. However, it has been shown that astrocytes alone are not able to sustain an efficient inflammatory response, for which microglia-derived TNF and other factors are required [103]. Thus, it is likely that microglial cells, the first to be activated after injury, represent the main source of TNF in the CNS, which is released to trigger astroglial pro-inflammatory activation [72,97]. Reactive astrocytes, as final effector cells, gain the ability to self-maintain their pro-inflammatory state by further releasing TNF, leading to long-lasting detrimental effects [102]. To close the circle, reactive astrocytes are able to modulate microglial activity, through inhibition of microglial TNF expression and release [104,105]. This process is essential to maintain the right kind of inflammatory response against brain damage, but it is often dysregulated in chronic disorders leading to a microglial switch toward detrimental phenotypes, contributing to the development of an extensive secondary injury [106].

In parallel to pro-inflammatory effects, tmTNF-mediated activation of microglial TNFR2 has been demonstrated to be essential for preserving the protective functions of these cells. Consistently, microglia-specific TNFR2 knockout mice display early onset of EAE and increased infiltration of pro-inflammatory immune cells from the periphery [107]. In this respect, it has been shown that TNFR2 regulates the expression of pro-regenerative and neuroprotective factors in microglial cells, including granulocyte colony-stimulating factor (gCSF) and IL-10 [108]. However, it is important to point out that the protective functions of TNF signaling in microglia have been greatly overlooked with respect to the inflammatory ones. Thus, further studies are needed to fully understand the mechanisms regulating the balance between TNFR1 and TNFR2 pathways in microglia in order to limit the detrimental immune responses without blocking the regenerative ones.

### 4.2. Neuronal Plasticity

The best documented role of microglia in controlling neuronal functions comes from the analysis of TNF effects on synaptic connectivity [109]. However, little is known about the action on synaptic function of TNF carried in microglial EVs.

TNF released by microglia has an important role in regulating synaptic plasticity [110]. Specifically, it controls a process called synaptic scaling, i.e., the adjustment of synaptic strength in response to prolonged changes in the electrical activity of neurons [110,111]. Indeed, a reduction of glutamate transmission increases microglial TNF release, which promotes the expression of AMPA glutamate receptors in neurons. Conversely, increased extracellular glutamate concentration inhibits TNF release from microglia, additional glutamate receptor expression, and declines neuronal activity [111,112,113]. The increase of AMPA receptor GluR1 subunit expression does not occur at mRNA level, but this is controlled by TNF at post-transcriptional level [114]. Subsequent studies revealed that TNF facilitates the trafficking and membrane insertion of AMPA receptors at the neuron surface, which are crucial for the homeostatic synaptic plasticity. Specifically, hippocampal neurons exposed to TNF increase surface expression of GluR1 subunit through modulation of NF-κB and acid sphingomyelinase pathways [115].

TNF not only controls homeostatic synaptic activity, but also induces neurotoxicity via autocrine/paracrine loops involving other endogenous mediators. First, TNF activates TNFR1 on microglia, amplifying its production and release [95]. Second, microglia-derived TNF activates TNFR1 expressed on astrocytes, allowing glutamate release from the glial cells. This, in turn, activates its specific receptors, including the metabotropic mGluR2 receptor on microglia, potentiating microglial TNF production and affecting synaptic transmission [110]. ATP, released by microglia concurrently with TNF, contributes to TNF-mediated neuronal damage by inducing a prolonged activation of microglial P2X7 receptor and release of both IL-1β and TNF inflammatory cytokines. In addition, both microglial TNF and ATP trigger adjacent astrocytes to release additional ATP, that amplifies microglia response and promotes astroglial release of glutamate, aggravating neuronal dysfunction [110]. Moreover, TNF mediates neuronal death by increasing extracellular levels of the excitotoxic transmitter glutamate and excessive AMPA receptor activation via downregulation of the astrocytic glutamate transporter EAAT2/GLT1 [116]. The effects of TNF on N-methyl-D-aspartate receptors (NMDARs) trafficking are less characterized. However, it has been demonstrated that, in hippocampal neurons, TNF increases the expression of the NR1 subunit of NMDAR and its specific clustering into lipid rafts [117]. Accordingly, treatment of human neuronal cultures with competitive (2-APV) and noncompetitive (MK-801) NMDA receptor antagonists reduced the glutamate neurotoxicity induced by TNF [118].

Furthermore, TNF also induces neuronal death by caspase activation and apoptosis induction, as demonstrated in cultured cerebellar neurons exposed to microglia conditioned medium, containing both solTNF and TNF-carrying vesicles [119]. The same authors showed that microglia release FasL, the death receptor ligand, which further potentiates TNF neurotoxicity after mGluR2 stimulation [119]. Similar deleterious effects were observed in primary cultured neurons exposed to the conditioned medium of BV-2 microglia treated with quinolinic acid (QA), a toxic agent increasing TNF production through NF-κB pathway [120]. Other studies showed that TNF is a potent inducer of oxidative stress in the CNS, that inhibits glutamate uptake by affecting glutamate transporters activity in astrocytes contributing to TNF-induced neuronal damage [121]. Of note, the neurotoxic effect mediated by TNF is dose-dependent. High concentration of TNF potentiates AMPA-induced neuronal death (through TNFR1 activation), whereas decreasing TNF dose results in neuroprotection (through TNFR2 activation) [122,123].

Another mechanism by which TNF promotes neurotoxicity is the inhibition of GABAergic transmission. In vitro studies in cultured rat and mouse hippocampal neurons indicate that TNF treatment induces a rapid and persistent decrease of inhibitory synaptic strength as well as a downregulation of cell surface levels of GABA_A_ receptors [124]. This results in excitatory/inhibitory unbalance, excessive entry of calcium through AMPA receptors and excitotoxic neuronal death. Consistently, microglial TNF increases the seizure susceptibility and epileptiform bursting in hippocampal slices of 2,4,6-trinitrobenzene sulfonic acid (TNBS)-treated animals, a model of peripheral inflammation [125]. This deleterious process is amplified by dying neurons, which in turn activate microglia and TNF production.

Similar alterations of synaptic transmission are induced by microglial EVs, which potentiate excitatory transmission while inhibiting release of the inhibitory transmitter GABA in cultures, brain slices and in the visual cortex [126,127]. However, such alterations were not attributed to the TNF cargo of microglial EVs. Specifically, Antonucci and colleagues showed that the stimulation of excitatory transmission by microglial EVs is due to the capacity of EVs to increase sphingolipid metabolism in neurons. Increased sphingosine and sphingosine 1 phosphate (S1P) synthesis enhances presynaptic release probability [126] and increases the number of synaptic vesicles in the ready releasable pool [128], respectively, at excitatory terminals. A subsequent study revealed that inhibition of presynaptic GABA release induced by microglial EVs occurs through modulation of the endocannabinoid system [129]. However, whether the TNF cargo of EVs released by reactive microglia may induce post-synaptic changes and contribute to excitatory-inhibitory unbalance remains to be explored.

So far, only a single study implicated the TNF cargo of microglial EVs in neuronal dysfunction. Specifically, it has been shown that exposure of murine microglial cell line BV2 to α-synuclein, to mimic the neurodegenerative environment associated to PD, increases the secretion of EVs enriched in TNF and major histocompatibility complex II (MHCII) molecules and promotes neuronal apoptosis [78]. In line with this study, parkinsonian patients were described to show an elevated number of TNF immunoreactive glial cells in the substantia nigra and high expression of TNF receptors in cell bodies and processes of most dopaminergic neurons [130]. However, microglial TNF seems to exert a dual function in PD, promoting degeneration in the striatum but supporting neuronal survival in the hippocampus [131]. This is consistent with a previous report by Rousselet et al. showing that TNF does not mediate the death of dopaminergic neurons but slightly alters their survival [132].

Besides PD, important roles of microglial TNF have been described in several other neuropathological contexts, such as AD, stress, ischemia, schizophrenia, and bipolar disorder [133]. Like in PD, microglial TNF plays a dual action in AD. It has been demonstrated that overexpression of TNF signaling enhances Aβ-induced pathology and learning and memory deficits [134], while TNF inhibition reduces cognitive impairment [135]. These findings suggest that targeting TNF signaling through block of its receptors may preserve neurons during AD pathology. Indeed, deletion of TNFR1 gene reduces plaque deposition and improves cognitive deficits in an AD mouse model [28]. Consistently, the administration of TNF pharmacological inhibitors or neutralizing antibodies decreases the activation of microglia, Aβ load, plaque formation and tau phosphorylation in AD mice [136,137]. On the other hand, TNF protects against glutamate, free radical, and Aβ toxicity in neurons in primary cultures [138] and reduces amyloid- and tau-related pathology in hippocampal neurons by activating TNFR2 [139]. In addition, in human neuronal cells TNF induces production of bcl-2, a molecule known to downregulate neuronal apoptosis [140].

Elevated levels of TNF were found in hippocampal microglia after acute stress and were shown to correlate with working memory deficits. Interestingly, treatment of stressed mice with the TNF inhibitor etanercept rescued such defects in accordance with a reduction in hippocampal TNF [141].

A dramatic upregulation of microglial TNF has been found in animal models of cerebral ischemia [142], specifically at the onset of neuronal cell death [143]. Studies examining cell death and survival following an ischemic insult showed that TNF production by microglia and the neuronal expression pattern of TNF receptors are determinant for neuronal death or survival [144,145,146]. Importantly, it has been demonstrated that TNF induces neurons to be resistant to a subsequent ischemic insult [147,148], reduces the size of the cortical infarcts and behavioral deficits, and modulates inflammatory responses [144].

The action of TNF can be influenced by environmental factors, such as exercise. Indeed, chronic exercise ameliorates cognitive impairment in wild-type mice by reducing the expression of hippocampal TNF and increasing the expression of TNFR1 and TNFR2. These benefits included reduced errors and improvements in spatial learning and spatial memory deficits in the Morris water maze [149]. However, the effect of exercise on cognitive function via TNFR1 and TNFR2 pathways remains unknown.

Given “beneficial” and “deleterious” functions of microglia and TNF in neurodegenerative diseases, the clinical application of anti-TNF therapies remains very difficult. However, an in-depth understanding of the effects of microglial TNF on synaptic functions and of the mechanisms underlying its actions may provide important insights for the development of novel therapeutic strategies aimed to limit neuronal loss and to improve cognitive deficits in patients.

### 4.3. Remyelination

A common feature of a diverse group of neurodegenerative diseases, from MS to psychiatric disorders and brain injuries, is demyelination, namely the damage to the myelin sheath. Myelin is the lipidic layer produced by oligodendrocytes which, enwrapping neuronal axons, allows the efficient action potential propagation by saltatory conduction within the CNS and provides mechanical and trophic support to neurons [150]. Myelin loss contributes to axonal degeneration and to long-lasting cognitive and motor disability that typically characterize later disease stages [151]. In response to axonal demyelination, oligodendrocyte precursor cells (OPCs), that are still present in the adult CNS tissue, proliferate, migrate at injury borders, and resume their differentiation program in order to replace degenerating oligodendrocytes with new myelinating cells [152,153].

Microglia heavily influence the pro-remyelinating attempts promoted by OPCs surrounding myelin lesions [154]. Indeed, by phagocytosing myelin debris and secreting trophic factors, microglia contribute to establish the permissive environment for efficient remyelination [154]. On the contrary, the impairment of these microglial functions, often paralleled by the acquisition of a pro-inflammatory phenotype, fatally leads to remyelination failure [155,156,157]. Of great relevance, selective pharmacological inhibition of solTNF was shown to promote remyelination through an increased clearance of myelin debris by microglia, suggesting that local release of solTNF represents an inhibitory factor counteracting myelin regeneration [158].

A very recent paper showed that TNF released by microglial cells, besides being fundamental for efficient clearance and degradation of myelin debris by phagocytes, it is also required for the generation of new myelinating cells within demyelinated areas, suggesting direct effects of this cytokine on OPCs [159]. This is in line with previous findings showing that TNF is able to finely regulate oligodendroglial functions, albeit its specific role still remains controversial. Historically, TNF was defined as a toxic molecule promoting death of adult human oligodendrocytes via JNK-3 activation followed by mitochondrial dysfunction [160]. A more recent study supported a role for TNF in mediating oligodendrocyte disruption occurring in patients and experimental models of MS, with a novel mechanism involving RIPK-1-mediated necroptosis [161].

Apparently in contrast with the aforementioned results, genetic ablation of TNF resulted in exacerbation of neurological deficits and augmentation of neuroinflammation and demyelination in the EAE mouse model. Of note, these deleterious effects were rescued by exogenous TNF administration [162], highlighting the protective function of TNF. The beneficial role of TNF was further confirmed by another elegant study showing delayed remyelination, due to reduced OPC proliferation and maturation, in TNF knockout mice undergoing cuprizone-induced demyelination [14].

The understanding of such opposing effects of TNF on oligodendrocytes [14] has increased thanks to subsequent studies highlighting the dichotomy between solTNF and tmTNF and between their two TNF receptor subtypes. In particular, MS and EAE have been associated with harmful effects of solTNF via TNFR1 [163,164,165], while activation of oligodendroglial tmTNF/TNFR2 axis is capable to sustain oligodendrocyte maturation and remyelination [14,15] and to modulate the local inflammatory milieu by limiting detrimental microglial activation and immune cell infiltration from the blood circulation [166].

Of note, TNF has been proved to be a key component of microglia-derived EVs involved in the astrocyte harmful conversion into oligo-toxic cells both in vitro and following lysolecithin-induced focal demyelination in vivo [72]. The importance of TNF, as protein cargo of microglial EVs, in the acquisition of detrimental astroglial functions was shown by the reduction of astrocytic pro-inflammatory response upon EV-carried TNF inactivation through etanercept co-administration [72]. Interestingly, TNF signaling may be directly involved also in astrocyte toxic effects on OPCs. Accordingly, astrocyte-derived TNF was demonstrated to inhibit oligodendrocyte differentiation and survival via TNFR1 stimulation [167]. However, given that the main source of TNF in both CNS and mixed glial cultures are probably microglial cells (see Section 4.1), in this context astrocytes may act by increasing oligodendrocyte sensibility to microglial TNF by promoting the upregulation of oligodendroglial TNFR1 [164]. Interestingly, this deleterious effect seems to require direct cell–cell contact, rather than soluble factors, as it can be prevented by physically separating astrocytes and oligodendrocytes in co-culture with a specific system avoiding direct cell interactions [164].

Intriguingly, TNF levels are dramatically decreased, but still detectable, in EVs derived from inflammatory microglia co-cultured with immunosuppressive mesenchymal stem cells (MSC-EVs), which do not cause harmful astrocyte transformation and instead promote oligodendrocyte maturation [72]. Based on this, it could be hypothesized that tmTNF, the isoform preferentially packed into microglial EVs (see Section 3), is able to promote different effects on target cells in a concentration-dependent manner. Low levels, i.e., those present in MSC-EVs, are able to selectively activate TNFR2 receptors, for which affinity values are higher [11], contributing to regenerative effects. On the contrary, high tmTNF levels carried by inflammatory EVs can activate also TNFR1, for which the affinity is low [11], triggering harmful responses. Thus, restoring the optimal concentration of TNF carried in microglial EVs may represent a good strategy to exploit the beneficial effects of TNF on myelinating cells.

## 5. Concluding Remarks and Future Perspectives

Activated microglial cells can release TNF through conventional ER-Golgi dependent or unconventional EVs-mediated secretory pathways in response to different stimuli. Accumulating evidence highlights the powerful but often controversial effects exerted by this cytokine on CNS cells. The dichotomous role of TNF in orchestrating different CNS processes, ranging from neuroinflammation and neurodegeneration to myelin repair, probably represents the main reason why TNF-targeting drugs have been proved to be largely unsuccessful for the treatment of neurological disorders [37,38,168,169].

Importantly, recent findings provide new insights on the mechanisms of TNF release and signaling that may be helpful for the development of novel therapeutic approaches. In this respect, the first thing to consider is the existence of two different isoforms, solTNF and tmTNF, which generally induce opposing effects by activating distinct TNFR1 and TNFR2 receptors [11]. A general rule that can be extrapolated from current literature is that an appropriate therapeutic approach should consist in selective inhibition of solTNF/TNFR1 signaling, which is responsible for detrimental effects, while preserving the functionality of tmTNF/TNFR2 axis, which underlies regenerative responses. Accordingly, selective blockade of solTNF by genetic ablation or treatment with the soluble inhibitor XPro1595 resulted in disease amelioration in experimental models of MS [158,170] and ischemic stroke [171,172]. Another intriguing approach is represented by the use of selective TNFR2 agonists, which showed neuroprotective effects in a model of NMDA-induced acute neurodegeneration [173] and ameliorated motor and cognitive symptoms in EAE mice by simultaneously suppressing CNS autoimmunity and promoting remyelination [174].

Besides directly targeting TNF signaling pathways with specific drugs, the mechanism of cytokine release may also represent an important target for an upstream modulation of TNF levels. Of note, unconventional release of TNF via EVs in response to ATP stimulation appears to be the prevalent mechanism of TNF secretion from activated microglia under disease conditions. Thus, blocking the shedding of microglial EVs by using pharmacological inhibitors [175] might reduce pathological TNF levels, counteracting the harmful effects of this cytokine. However, it is to be considered that this strategy also inevitably loses the beneficial contribution of TNF to tissue repair.

Finally, the phenotype of TNF-donor cells seems to pivotally determine TNF levels packaged into EVs, which in turn impact the final effects of EVs on target cells. Accordingly, EVs produced by inflammatory microglia contain high amounts of TNF and induce detrimental astrocyte conversion and demyelination [72,76]. On the contrary, microglia co-cultured with immunosuppressive MSCs produce EVs carrying low levels of TNF. This abolishes the harmful effects of microglial EVs and, instead, promotes myelin repair [72]. Thus, redirecting inflammatory microglia into pro-resolving cells clearly emerges as an effective strategy to restore the correct balance of vesicular TNF cargo, reducing inflammatory damage and fostering tissue regeneration by EVs. This is in line with the diffused opinion that reprogramming microglial cells towards beneficial functions, rather than generally suppressing their activation, might sustain repair processes efficiently [176].
